# Thermodynamics of lipid multi-lamellar vesicles in presence of sterols at high hydrostatic pressure

**DOI:** 10.1038/s41598-017-15582-4

**Published:** 2017-11-10

**Authors:** J. Peters, J. Marion, F. J. Becher, M. Trapp, T. Gutberlet, D. J. Bicout, T. Heimburg

**Affiliations:** 1 Univ. Grenoble Alpes, LiPhy, 140 Rue de la Physique, 38402 Saint-Martin-d’Hères, France; 2 Institut Laue-Langevin, 71 avenue des Martyrs, CS 20156, 38042 Grenoble cedex 9, France; 3 Niels Bohr Institute, University of Copenhagen, Blegdamsvej 17, 2100 Copenhagen, Denmark; 4 Helmholtz-Zentrum Berlin für Materialien und Energie, Lise-Meitner Campus, Hahn-Meitner-Platz 1, 14109 Berlin, Germany; 5 Jülich Centre for Neutron Science (JCNS) at Heinz Maier-Leibnitz Zentrum (MLZ), Forschungszentrum Jülich GmbH, Lichtenbergstr. 1, 85748 Garching, Germany; 6 Biomathématiques et épidémiologie, EPSP – TIMC-IMAG, UMR CNRS 5525, Université Grenoble Alpes, VetAgro Sup Lyon, 69280 Marcy l’Etoile, France; 7 Present Address: Department of Chemistry, University of Cambridge, Lensfield Rd, Cambridge, CB2 1EW UK

## Abstract

We compared the effect of cholesterol at different concentration on the phase behaviour of DMPC (1,2-dimyristoyl-*sn*-glycero-3-phosphocholine) multilamellar vesicles. We used pressure perturbation differential scanning calorimetry (PPC) that studies a system on the whole by giving access to relevant thermodynamic quantities, and elastic incoherent neutron scattering (EINS) that probes local motions of a system at the atomic level by allowing extraction of dynamical parameters. PPC revealed that the volume expansion coefficient of DMPC and DMPC/Cholesterol samples with 13 and 25 mol% cholesterol is a linear function of the heat capacity measured by differential scanning calorimetry. Neutron backscattering spectroscopy showed that the mean square displacements of H atoms do exhibit an increase with temperature and a decrease under increasing pressure. Cholesterol added at concentrations of 25 and 50 mol% led to suppression of the main phase transition. Taking advantage of these results, the present study aims (i) to show that calorimetry and EINS using the Bicout and Zaccai model equally permit to get access to thermodynamic quantities characterizing pure DMPC and DMPC/cholesterol mixtures, thus directly confirming the theoretical method, and (ii) to validate our approach as function of temperature and of pressure, as both are equally important and complementary thermodynamic variables.

## Introduction

The system under investigation is a lipid model membrane composed by 1,2-dimyristoyl-*sn*-glycero-3-phosphocholine (DMPC), in presence or not of cholesterol. Not only are lipids and membranes very important components of the cells, they are also among the bio-molecules the most sensitive to high pressure^1^. Cholesterol accounts for up to 50% of the lipid content in mammalian cell membranes and has the remarkable effect of making the membranes stiffer, but maintaining them in a liquid-ordered phase (l_o_) and thus functional under normal physiological conditions. The stiffness, inversely related to the flexibility of the membrane, influences its thermodynamic state. Indeed, above certain cholesterol concentrations, which depend on the specific lipid kind, the pre-transition between the gel and the ripple phase first disappears. In fact, the pre-transition does not occur in all lipids and is almost special to the fully saturated ones, which are not biologically relevant. Then the main phase or melting transition between the ripple and the fluid phase is slightly shifted to a lower temperature (about 1.5 °C for 1,2-dipalmitoyl-*sn*-glycero-3-phosphocholine (DPPC)), damped and broadened and finally vanishes^2^. For high cholesterol contents, a new phase was postulated by Linseisen *et al*.^3^, the liquid-ordered phase, where the membrane molecules show a positional disorder and a high lateral mobility, which is typical for a liquid phase. On the other hand, due to the ordering effect of cholesterol, the chains have a substantial degree of conformational order. Therefore, it makes the bilayer tighter, what is one of the requests for the correct functioning of a cell membrane allowing to create chemical gradients and to harvest the energy^4^. Hence, introducing cholesterol into the membranes has a similar effect as decreasing temperature.

The variation of the thermodynamic variables in presence of cholesterol in membranes is crucial to understand the physics and mechanics underlying the function of biomembranes and the role played by cholesterol within. Pressure perturbation differential scanning calorimetry (PPC) gives direct access to various thermodynamic values as enthalpy H, heat capacity c_p_ or volume expansion coefficient α_v_^5–7^. In contrary, elastic incoherent neutron scattering (EINS) allows the observation of an average of the atomic trajectories of local motions within the time and space windows defined by the energy and spatial resolution of the neutron spectrometer in use.

The idea to study the effect of cholesterol addition to lipids is not new and was already investigated by several methods like FRAP^8^, Raman scattering^9^ or FTIR^10^ to name only a few. Halstenberg *et al*.^11^ used differential scanning calorimetry (DSC) to shed light on the correlation between heat capacity and compressibility close to the chain-melting transition, where volume and inner energy are proportional functions of the temperature. NMR can be used in order to extract structural parameters of the lipid/cholesterol system and results can be compared to other techniques such as neutron or x-ray diffraction^12^. Not only structural but also dynamical processes like lateral diffusion^13,14^ in the membrane or raft formation^15^ can also be a topic for NMR investigations. Méléard *et al*.^16^ used solid-state NMR and found an absence of critical behaviour in the bending modulus of DMPC-cholesterol membranes at high cholesterol content. Another investigation done by McMullen *et al*.^17^ with DSC concerns the phase behaviour of DPPC bilayers in presence of sterols. The authors determined alkyl chain length-dependent effects, with both the transition temperature and cooperativity decreasing more dramatically with progressively shorter lipid side chains. Mabrey *et al*.^18^ studied the morphology of DPPC-cholesterol mixed monolayers formed on water surface by various methods and found that the fluidic DPPC monolayer changed to a condensed rigid monolayer due to the condensation effect of cholesterol.

Extensive effort has also been laid on the investigation of the phase diagram of lipid/cholesterol^8,19,20^ by molecular simulations, the structural changes induced by adding cholesterol^21^ and domain formation^22^. In an early work using quasi-elastic neutron scattering (QENS) Gliss *et al*.^23^ have investigated the effect of different cholesterol concentrations on the dynamics of oriented stacks of DPPC in the liquid-ordered phase. A strong anisotropy between in-plane and out-of-plane motion was found and the authors concluded that cholesterol can move between the two leaflets of the lipid bilayer. The study was extended to lanosterol and ergosterol by Endress and co-workers^24^ but only cholesterol showed the flip-flop motion. In a recent investigation Busch *et al*.^25^ found a decreased mobility of DMPC in the presence of cholesterol on a 900 ps timescale whereas all other additives investigated showed no significant effect on the lipid dynamics. In a separate study by Armstrong *et al*.^26^ inelastic neutron scattering was employed to investigate collective dynamics of the lipid/cholesterol system. There was a co-existence of excitations found and the study concluded that this was caused by coexisting nanodomains of gel, fluid and l_o_ domains in the membrane.

Combining pressure and temperature calorimetry and elastic incoherent neutron scattering experiments allows a direct comparison of obtained results by the two techniques, which probe very different time scales, to obtain complementary results on the thermodynamic values describing the system and permits a better understanding of biomembrane mechanics at local molecular level.

## Results

### Calorimetry

**Figure 1:**
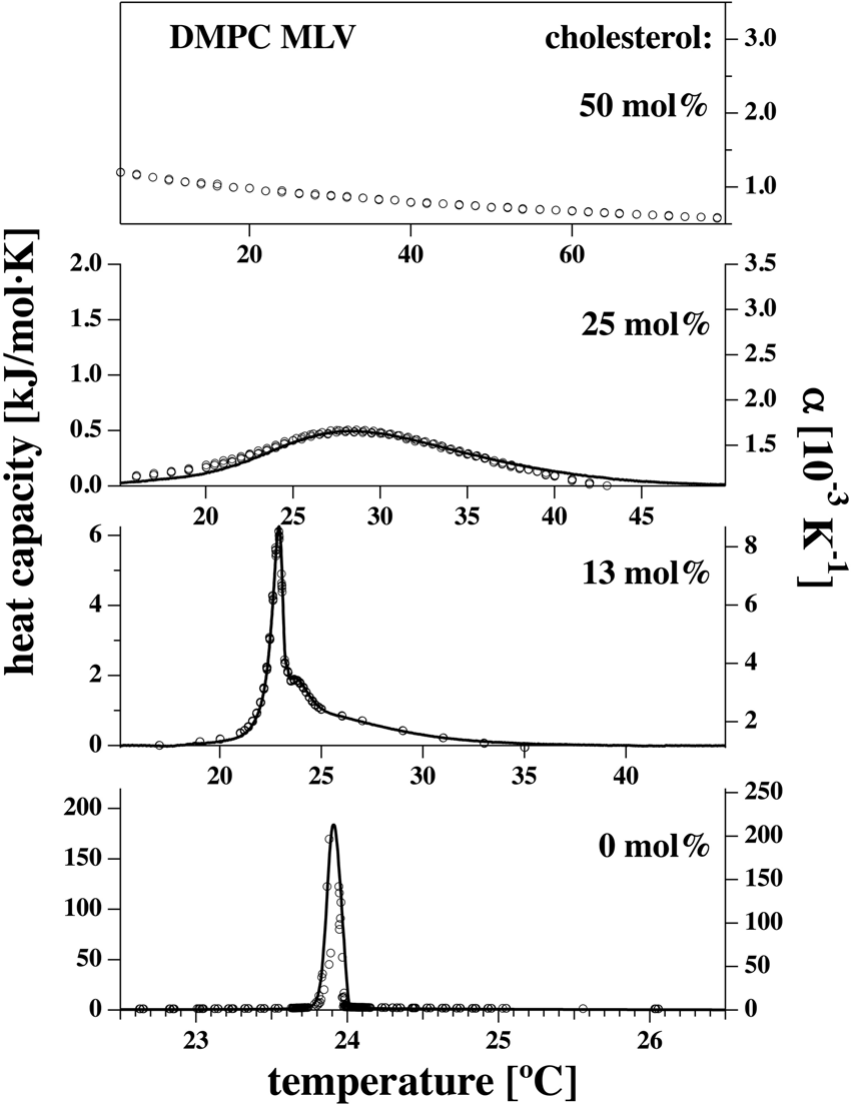
Excess heat capacity profiles Δc_p_ (solid lines) and volume expansion coefficients α_V_ (open circles) for DMPC MLVs in the presence of 0 mol%, 13 mol%, 25 mol% and 50 mol% cholesterol (from bottom to top). For 50 mol%, the melting profile was so much broadened that no heat capacity anomaly could be identified. The volume expansion coefficient does not display an anomaly, either. The α_V_ values were corrected for the baseline of pure water but plotted without subtracting the value of the volume expansion outside of the phase transition.

In Fig. 1 both the excess heat capacity Δc_p_ (from DSC measurements) and the volume expansion coefficient α_v_ (from PPC) for DMPC multilamellar vesicles (MLVs) in the absence and the presence of cholesterol, using 0 mol%, 13 mol%, 25 mol% and 50 mol% of cholesterol are shown. We find that both functions display very similar temperature dependence. While pure DMPC MLVs present a very sharp transition, the profile is widened in the presence of 13 mol% cholesterol, and even more broadened in the presence of 25 mol% cholesterol. We could not detect any distinct peak or anomaly in the presence of 50 mol% cholesterol (here, only the volume expansion coefficient data are shown). This either indicates that the transition does not take place or, more likely, that the profile is so broad that one cannot reasonably determine a baseline. The peak maxima correspond to 23.91 °C for 0 mol% of cholesterol, to 22.90 °C for 13 mol% and 28.27 °C at 25 mol%. These results are in agreement with^8,27–29^. In^8,27^ it is also evidenced that no phase transition is detectable at 50 mol% cholesterol and in refs^27,29^ it is shown that the phase transition becomes very broad at 25 mol% cholesterol.

Due to the uncertainty of the baseline with very broad profiles, integrals of heat capacity (transition enthalpy) and volume expansion coefficients (transition volume) should generally be interpreted with caution and are therefore not shown here. For the very sharp DMPC MLV data, we found that the total enthalpy change including pre-transition was ΔH = 29.6 kJ/mol, whereof 4.7 kJ/mol were due to the pre-transition, and 24.9 kJ/mol due to the main transition. From the data in Fig. 1 we find empirically that

1 $$\frac{d{\rm{\Delta }}V(T)}{dT}=\gamma \frac{d{\rm{\Delta }}H(T)}{dT}.$$

This relation has been confirmed in molecular dynamics simulations^30^ and similar behavior has been described in the literature for van-der-Waals liquids and glasses. However, it does not represent a theoretical derivation based on first principles.

Here we determine γ = 7.86·10^−4^ ml/J for DMPC MLV, γ = 8.14·10^−4^ ml/J for DMPC MLV in the presence of 13 mol% cholesterol, and γ = 8.31·10^−4^ ml/J for DMPC MLV in the presence of 25 mol% cholesterol (see Table 1). The experimental error is less than 2%^6^. These values are very close to those reported from pressure calorimetry^6^ for DMPC vesicles to be γ = (7.81 ± 0.11) 10^−4^ ml/J and for DPPC vesicles to be γ = (7.86 ± 0.11) 10^−4^ ml/J. Within experimental error, the present values determined with pressure perturbation calorimetry are identical for the different lipid systems. Further, they are within errors the same for all samples and not influenced by the presence of cholesterol. Based on these values the pressure dependence of the enthalpy in the presence and absence of cholesterol can be determined.

**Table 1:** Proportionality factor γ between excess heat capacity Δc_p_ and excess volume expansion coefficient α_v_. The values for 0 mol%, 13 mol% and 25 mol% cholesterol were determined from the data in Fig. 1.

DMPC/cholesterol	0 mol %	13 mol %	25 mol %	50 mol %
γ[10^−4^ ml/J]	7.86 ± 0.24	8.14 ± 0.41	8.31 ± 0.42	—

Using eqs (6) and (7) in the Materials and Methods section, we obtain a shift in the transition temperature *T*_*m*_ of 6.96 °C with Δp = 300 bar, and 13.92 °C with Δp = 600 bar, which is valid for DMPC and DMPC/cholesterol mixtures within the given accuracy. This corresponds to 43.1 bar/°C. Since the γ-factors are close to each other in the absence and the presence of cholesterol, the shift in *T*_*m*_ seems to be independent of cholesterol content. This is consistent with results presented in Trapp *et al*.^31^, where the transition temperature shifts were found to be 6 ± 2 °C at 300 bar and 12 ± 2 °C at 600 bar. It is interesting to note that the analysis of Ebel *et al*.^6^ also included that the calorimetric profiles got broader under pressure, but could be superimposed by a transformation ${T}^{\ast }=T/(1+\gamma {\rm{\Delta }}p)$ and ${\rm{\Delta }}{H}^{\ast }={\rm{\Delta }}H/(1+\gamma {\rm{\Delta }}p)$.

### Neutron scattering

**Figure 2:**
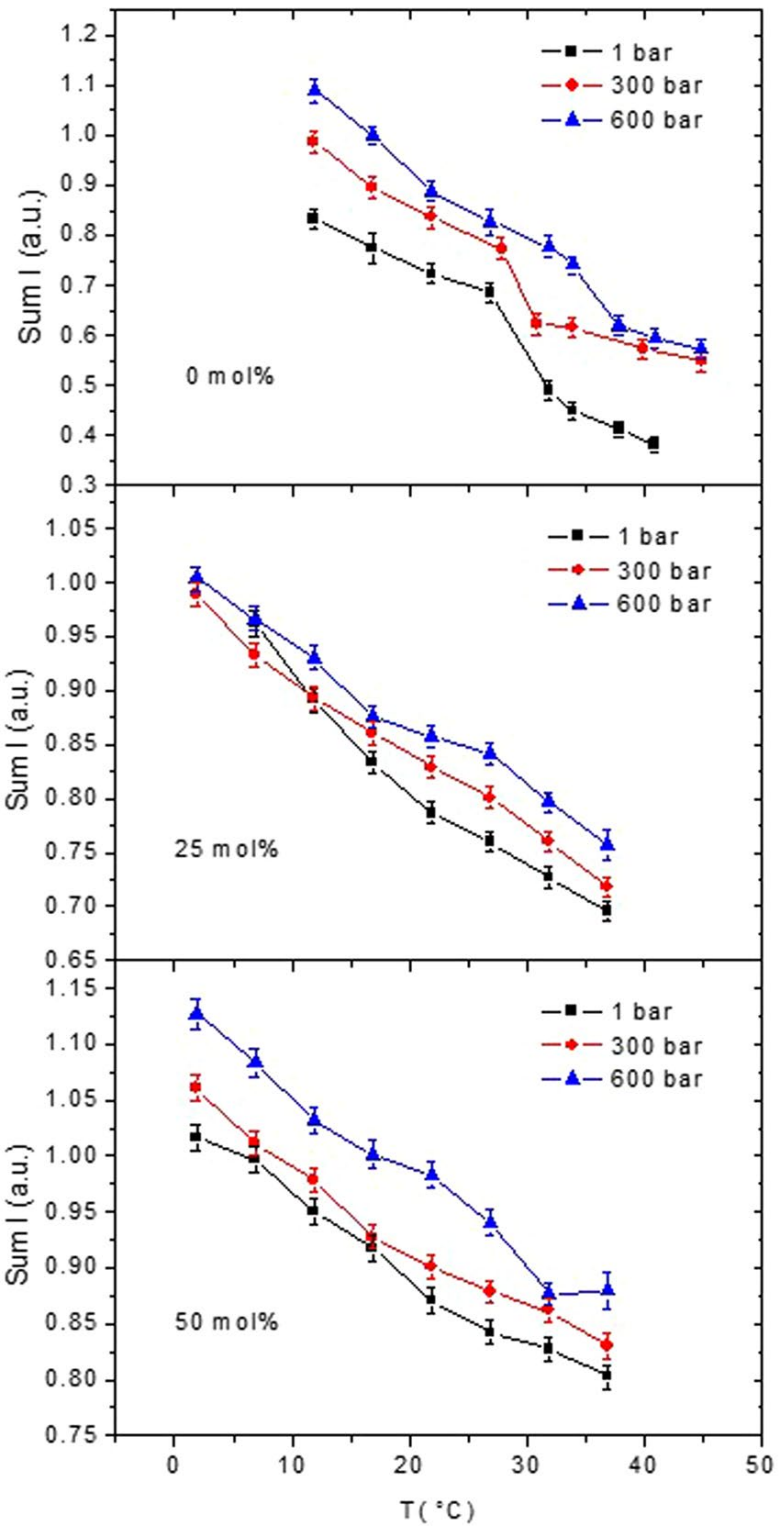
Summed elastic intensities of pure DMPC (reproduced from ref.^31^) and DMPC/cholesterol mixtures, rescaled for the total amount of lipids, measured on IN13. Data at atmospheric pressure are shown as black squares, data taken at 300 bar as red circles and data recorded at 600 bar as blue triangles. Data are summed over the complete Q-range available on IN13 (0.29 Å^−1^ < Q < 4.98 Å^−1^). Lines connecting the data points are guide to the eyes only.

Figure 2 represents the raw data of the elastic neutron intensities (corrected only for the incoming neutron flux) for pure DMPC (reproduced from ref.^31^) and the DMPC/cholesterol mixtures at 25 and 50 mol% cholesterol summed over all accessible scattering angles (corresponding to a range of 0.29 Å^−1^ < Q < 4.98 Å^−1^). In contrast to the MSD, where the Gaussian approximation^32,33^ has to be fulfilled, the integrated intensities are not restricted to the Q-region below 1.47 Å^−1^. The mixture with 13 mol% cholesterol was not measured due to experimental time constraints. In pure DMPC, the pre-transition is not detectable by EINS^34^, obviously because the thermal fluctuations induced by it are not sufficient to make a significant impact on the scattering signal, but the main phase transition appears as a change of slope in the MSD^31^. Similar to the DSC data the sterols wash out the effect of the phase transition, which is no longer visible for the cholesterol concentrations of the neutron scattering experiments at any applied pressure. (Fig. 1 confirms that the heat capacity anomaly is very small at 25 mol% cholesterol and not measureable at 50 mol% cholesterol). The intensities increase with pressure at a given temperature as expected, but the variations with pressure are bigger at higher cholesterol concentration.

**Figure 3:**
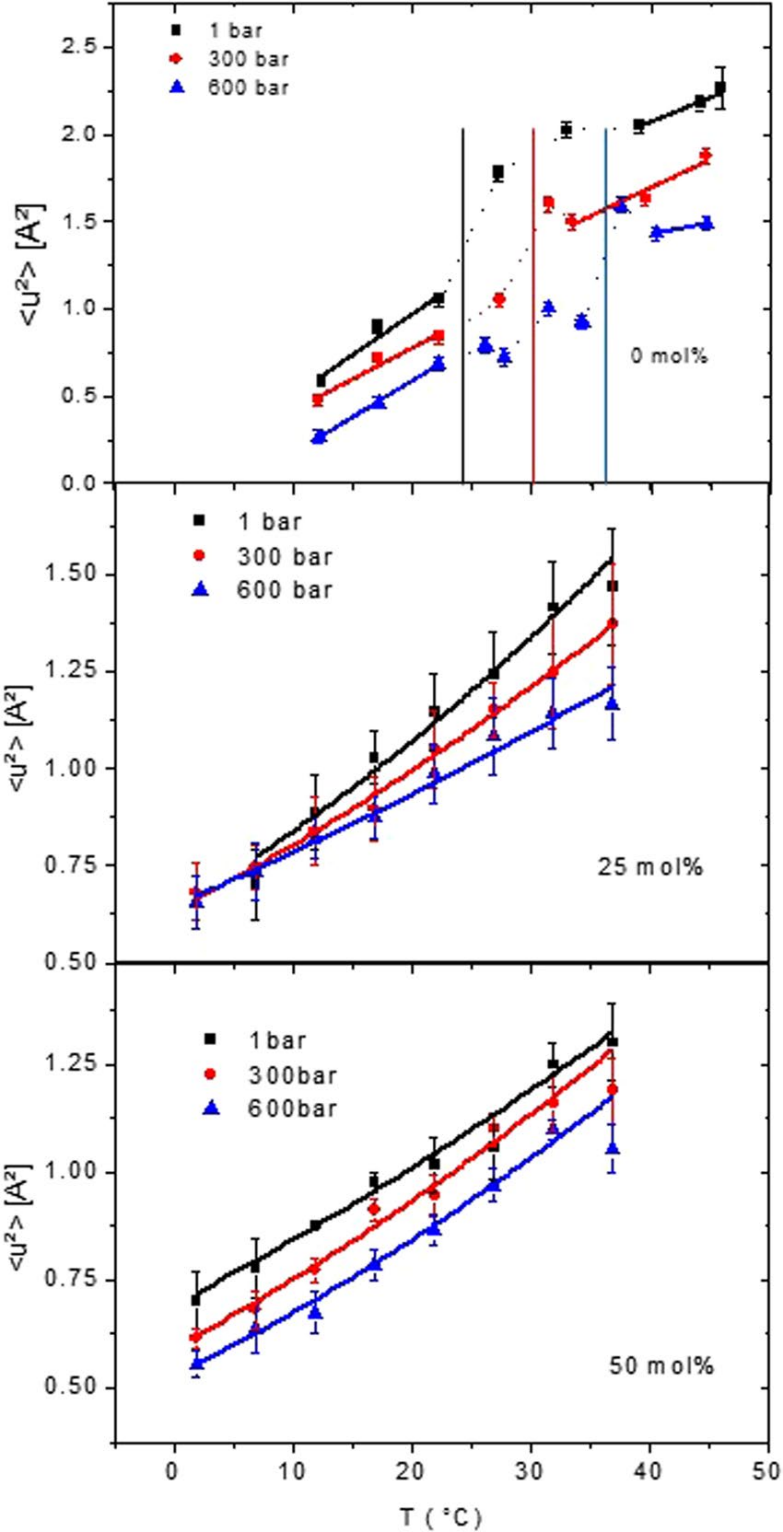
MSD of pure DMPC (reproduced from ref.^31^) and both DMPC/cholesterol mixtures calculated according to eq. (17) (without any polydispersity and C_α_(τ) = 0). Black squares represent data at atmospheric pressure, red circles correspond to 300 bar and blue triangles to 600 bar. The lines show the fits according to eq. (20) and the effective force constants are calculated with the help of eq. (21). For 0 mol% cholesterol, the dotted lines in the transition region are only guide to the eyes. The vertical lines mark the phase transition temperature at that pressure value.

The summed elastic intensities are inversely proportional to the root of the MSD^35^. The MSD are associated with the dynamic flexibility of the molecules and are a measure of the fluctuations in this temperature region, as explained in more details in a publication by M. Rheinstädter *et al*.^36^. Figure 3 represents the MSD of pure DMPC (reproduced from ref.^31^) and DMPC with the two different concentrations of cholesterol. Evaluation of the multiple scattering corrections for pure DMPC showed a negligible effect compared to self-shielding and absorption. The same was found for elastic scans of membrane systems by Busch *et al*.^37^. Therefore, for the DMPC/cholesterol mixtures no multiple scattering corrections were applied. As stated above, the main phase transition disappears in this temperature range and for both concentrations. However, the slopes of the MSD are decreasing with HHP in the case of the 25 mol% concentration and stay almost constant in the case of the 50 mol% concentration.

**Figure 4:**
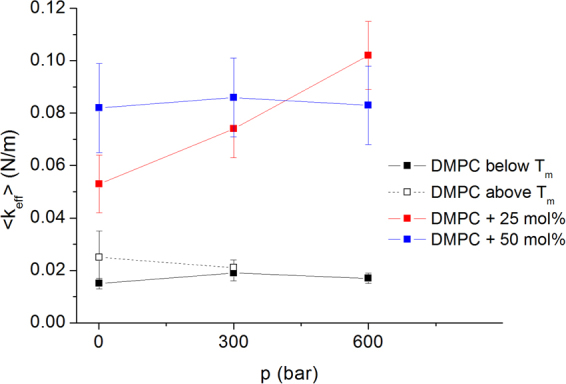
Effective force constants as function of pressure for DMPC and two different sterol concentrations. The values for DMPC are taken from ref.^31^.

To derive the force constants we applied the model of Bicout and Zaccai^38^, e.g. we fitted the MSD according to eq. (20) with a force constant defined by eq. (21), both given in the Materials and Methods section. The fit results are included in Fig. 3 and the corresponding effective force constants are given in Fig. 4 and Table 2 for pure DMPC and both sterol concentrations. The force constants are overall lower compared to typical values for biological membranes or proteins^39,40^, thus corresponding to a higher dynamic flexibility of the studied MLV as was already documented for pure lipids^41^. However, they are increased in presence of cholesterol compared to pure DMPC or pure DPPC where <k_eff_> is typically ≈0.015 N/m [extracted from ref.^42^], proofing that cholesterol stabilises the lipids. <k_eff_> is constant in pressure for pure DMPC, increasing with pressure for the lower sterol concentration of 25 mol% and staying again almost constant at the concentration of 50 mol%, but at a much higher value.

**Table 2:** Effective force constants calculated for DMPC + cholesterol at three HHP values and two different sterol concentrations. The values for DMPC are taken from^31^. We did not evaluate the force constant for DMPC above T_m_ at 600 bar, as we had only two experimental points for doing that.

Pressure (bar)	DMPC below T_m_ <k_eff_> [N/m]	DMPC above T_m_ <k_eff_> [N/m]	DMPC +25 mol% chol. <k_eff_> [N/m]	DMPC +50 mol% chol. <k_eff_> [N/m]
1	0.015 ± 0.002	0.025 ± 0.010	0.053 ± 0.011	0.082 ± 0.017
300	0.019 ± 0.003	0.021 ± 0.003	0.074 ± 0.011	0.086 ± 0.015
600	0.017 ± 0.002		0.102 ± 0.013	0.083 ± 0.015

## Discussion

It is well established that molecular dynamics play an as important role for the functioning of bio-systems as structure^43,44^. It can be influenced by external conditions such as temperature, pressure, hydration or pH values^45^. However, dynamics exist at very different timescales, including thermal fluctuations covering picoseconds to nanoseconds up to kinematics of enzymatic activity at the micro- to millisecond timescale or cell divisions at the second to minute scale. It is a longstanding debate to understand whether such motions and related relaxation processes are coupled over several orders of magnitude^46^, but recent studies point toward such understanding^44,47^.

Whereas it is possible to detect thermal local motions in all kind of matter, diffusional (translational or rotational) movements appear in biomolecules strongly depending on the state of hydration^48^. In addition, if the experimental technique gives access to very small energy transfers, one can observe collective vibrational excitations as well^47^. Some bio-systems undergo phase transitions, depending on the external conditions, which are accompanied by important structural rearrangements and fluctuations. E.g. in phospholipid-cholesterol mixtures the main phase transition is suppressed at concentrations of cholesterol above 33 mole % due to domain formation^49^. Also substitution of solvent from water to ethanol suppresses the main phase transition in phospholipid bilayers depending on pressure^50^. In fact, phase transitions or critical phenomena are characterized by fluctuations of all possible size and length scales. The correlation length tends to infinity at the transition then resulting in a scale invariance^51^. Therefore, around a phase transition, the underlying fluctuations are inherently connected to macroscopic thermodynamic parameters.

Therefore, dynamics fluctuations at molecular scale can be connected to thermodynamics at any time scale, as for instance at the sub-nanosecond scale probed typically in neutron scattering experiments. Moreover, as neutrons are a very weakly interacting probe, they monitor the unperturbed sample state, i.e. the experiment yields direct information on the spontaneous fluctuations in the sample, and this despite the fact that the neutrons interact with it^52^. As a general rule it holds that the larger the amplitude, the slower the motion and *vice versa*. Each atom is bound to other atoms and molecular sub-groups, thus it can execute small motions around its equilibrium position or diffuse together with the whole molecule or domain. Larger amplitudes of motion request more energy, hence they become more likely at higher temperature. Such a picture has been described as the conformational landscape of biomolecules for particular proteins^43^, where each sub-state is associated with a given free energy state, and we assume that similar conclusions apply for lipids as well. As long as the movements are restricted to vibrations around an equilibrium position, they are harmonic in the lowest order approximation, but when the atoms have sufficient energy to cross barriers between different sub-states the motions become necessarily non-harmonic. As the barrier crossing between two different potential wells requests a certain amount of energy, the transition between different sub-states is related to an energy exchange and therefore to the thermodynamics of the system. As this amount of energy lies above the energy corresponding to the lower state, we call it in the following excess energy.

To treat both, the fluctuations and the macroscopic quantities, mean field theory can be applied in a first approximation with the aim to validate it by comparing the outcome on two completely different time- and length scales (seconds’ scale compared to the sub-nanosecond dynamics) obtained through different techniques (calorimetric measurements versus incoherent neutron scattering techniques).

As function of temperature, it is already established for incoherent neutron scattering data that it is possible to describe a dynamical transition in terms of a free energy barrier ΔG^53^ including excess enthalpy and entropy changes, but such investigation has never been undertaken as function of pressure. However, temperature is only one important thermodynamic variable, and one cannot expect describing a system completely as long as the dependence on pressure is not taken into account^54^. Therefore, our approach uses a corresponding treatment to neutron scattering data and to DSC and PPC experiments to make the relation between them. The lipid phase transitions occur at much higher temperature than the dynamical transition, but as the picture of a cross over from one state to another, which requests free energy, still holds, the same kind of formalism can be applied. Moreover, we aim for a validation of the mean field approach used here. In particular, experiments probing the local dynamical behaviour of biomembranes under high hydrostatic pressure (HHP)^55,56^ applying neutron scattering are scarce. This fact is attributed to the experimental challenges combining this technique with lab based methods as PPC.

In a previous publication^31^ first results of pressure effects on the dynamic behaviour of MLVs of pure DMPC studied with elastic incoherent neutron scattering were presented. In the current study the phase behaviour of pure DMPC and DMPC/cholesterol mixed systems as a function of pressure and temperature is first characterised using calorimetry and second EINS. Applying pressure, the membrane order is increased and therefore the phase transition temperatures altered accordingly. EINS allowed to investigate the local dynamical response of the pure DMPC and mixed DMPC/cholesterol samples to pressure and to relate it to the thermodynamic quantities as the excess enthalpy and entropy changes complementary to the calorimetry data revealed.

The calorimetry data reveal a subsequent broadening of the heat capacity and the volume expansion with increasing amount of cholesterol. As the ratio γ between the volume change ΔV and the excess enthalpy change ΔH_0_ is only slightly increasing on increasing concentration of cholesterol a distinct pressure dependence of the excess enthalpy change on the presence of cholesterol is not visible. A similar result was reported by Ebel *et al*.^7^.

Concerning the neutron scattering technique, the addition of cholesterol in concentrations of 25 and 50 mol% to DMPC makes the phase transitions disappear in the MSD and flattens the corresponding curves. This permits fits of the MSD as function of temperature in presence of cholesterol according to eq. (20) with a force constant defined by eq. (21) in the Materials and Methods section and extracting thermodynamic quantities, as well as the changes in enthalpy and entropy with pressure (see Table 3).

**Table 3:** Thermodynamic parameters for DMPC + cholesterol at two different concentrations extracted from EINS. The absolute error on pressure is ± 30 bar.

DMPC + Cholesterol	P [Bar]	Δ*S* [J/Kmol]	Δ*H* [kJ/mol]
25 mol%	1	41.4 ± 4.6	16.6 ± 0.6
	300	38.5 ± 3.8	14.9 ± 0.5
	600	32.5 ± 3.5	12.4 ± 0.4
50 mol%	1	30.1 ± 5.1	12.4 ± 0.6
	300	40.5 ± 4.5	15.3 ± 0.5
	600	39.8 ± 4.3	15.5 ± 0.6

At 25 mol% of cholesterol content, both the total enthalpy, ΔH, and the entropy changes, ΔS, are positive, but decrease with pressure. This means that the rise of temperature induces an endothermic process increasing the disorder and dynamics of the system, but pressure counteracts these changes. This behaviour seems inversely related to the dependency of the effective force constant <k_eff_> on pressure. The increase of the force constant at 25 mol% is indeed the signature of a higher mechanical stability of the system when the pressure is going up.

At an increased amount of 50 mol% of cholesterol, the dependence on pressure remains unclear. On one hand the force constant (see Table 2) is higher at 1 bar compared to 25 mol% of sterols, confirming that cholesterol stiffens the vesicle membrane, but the value stays constant with pressure. This behaviour might indicate a stabilising effect at such high sterol content. On the other hand, the enthalpy and entropy changes rise until 300 bar, but then stay constant until 600 bar (Table 3). One should notice that the fact to find fluctuations of any size is only true close to phase transitions and we cannot determine accurately the concentration where the phase transition disappears in presence of cholesterol, it is to say where the system is no longer homogeneous, but phase separation might occur.

We can only speculate about the reasons for such behaviour, but we believe that it is likely related to the underlying phase transitions, as reported for instance by de Meyer *et al*.^20^: at 0 mol% the lipids are undergoing two phase transitions from the gel to the ripple and then to the fluid phase, where the second one has a significant impact on the dynamics. The lipids are rather tightly packed, and thus the force constant is not very sensitive to pressure for pure DMPC. As cholesterol fits in between the chains of the phospholipids, they create more space in-plane at 25 mol%^57^. At lower temperature (below 15 °C) the lipids are in the highly ordered lamellar crystalline phase, going then to the ripple or liquid-ordered phase to end up in the fluid phase around 30 °C^20^. These multiple phase transitions could provoke the changes in enthalpy, entropy and the effective force constant as found here. Finally, at 50 mol% of cholesterol, the MLVs are first in the lamellar crystalline phase passing directly into the liquid-ordered phase l_o_, which are both very tightly packed, thus less sensitive to pressure. The enthalpy and entropy changes are decreased with respect to the lower cholesterol concentration, therefore the transitions can be overcome at lower pressure to stay then nearly constant.

The total enthalpy change values taken from calorimetric experiments^28^ are for pure DMPC 29.6 kJ/mol, for 13 mol% 12.2 kJ/mol, for 25 mol% 7.5 kJ/mol and for 50 mol% 0 kJ/mol. These results are very similar to those given by Tamai *et al*.^27^. The values derived from neutron data are at the same order of magnitude at 25 mol%, but at 50 mol%, we find a similar value as at 25 mol% with neutrons, but nothing from calorimetry. However, due to the broadening of the peak, the baseline could not be reasonably determined, so that the disappearance of the enthalpy of the transition might be an artefact of this technique.

According to the Clausius-Clapeyron principle the melting points T_m_ of a membrane are linked to pressure through

2 $$\frac{d{T}_{m}}{dp}={T}_{m}\frac{{\rm{\Delta }}V}{{\rm{\Delta }}H}=\frac{{\rm{\Delta }}V}{{\rm{\Delta }}S}.$$

Using the data presented in ref.^31^, the volume change at ambient pressure is calculated to be 16,1 mL/mol for the transition from the compact ordered state to the disordered liquid state for pure DMPC. In the same way the volume change is calculated in presence of 25 mol% cholesterol at the three measured pressure points to obtain 11,2 mL/mol, 10,1 mL/mol and 8,4 mL/mol with rising pressure. The volume change is always smaller than in the sterol free MLV and is close to the value which can be estimated from the curves given in ref.^11^.

**Figure 5:**
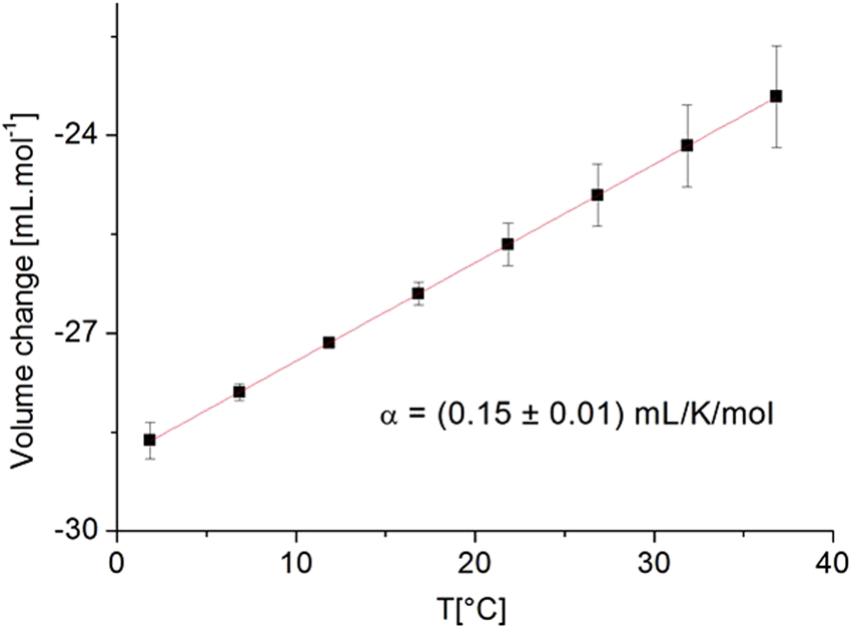
Volume change extracted for DMPC at 25 mol% of cholesterol. The line is a linear fit to the data in order to extract $\alpha =\frac{\partial \,{\rm{\Delta }}{V}_{{\rm{T}}}}{\partial T}$.

We also extracted the isothermal volume change from the slope of the equilibrium constant as function of pressure for the lower sterol content (the uncertainty about the results at 50 mol% do not allow a deeper analysis) according to eq. (22) and got negative values (see Table 3), i.e. a volume reduction with pressure at constant temperature in accordance with Le Chatelier’s principle^58^. The volume change is, however, smaller for higher temperature (−23.2 mL/mol at 37 °C instead of −28.7 mL/mol at 2 °C, see Fig. 5) as the two thermodynamic parameters have competing effects on it.

For pure DMPC in liquid-crystalline phase, e.g. above 24 °C, the thermal expansivity $\alpha =\frac{\partial {\rm{\Delta }}{V}_{T}}{\partial T}$ is given as (0.65 ± 0.11) mL/K/mol by^59^. In the system with 25 mol%, we obtain a value of α = (0.15 ± 0.01) mL/K/mol by ftting ΔV│_T_ as function of T (see Fig. 5), which is more than four times less compared to pure DMPC but in accordance with findings of ref.^11^ at this sterol content. Moreover, we fitted over the temperature range 7–37 °C corresponding to the regime where the lipids transit from the ripple to the liquid-crystalline phase, but the transition did exhibit no measurable influence on the volume change in presence of cholesterol. It should be noted that for all mixtures of lipids with cholesterol, the relation $\frac{dV(T)}{dT}=\gamma {c}_{p}$ was used with a constant γ value. It is in accordance with a recent publication by Pedersen *et al*.^30^ which suggested that these relations were not only valid in the transition region, but also in pure phases with a constant γ value.

The former discussion of the neutron scattering results confirms the validity of the mean field approach in this context. We have shown that it is possible to compare the parameters derived by both studies by considering the relevant length and time scales of the fluctuations accessible in DSC and neutron scattering which allows to extract further information about the phase transition.

**Figure 6:**
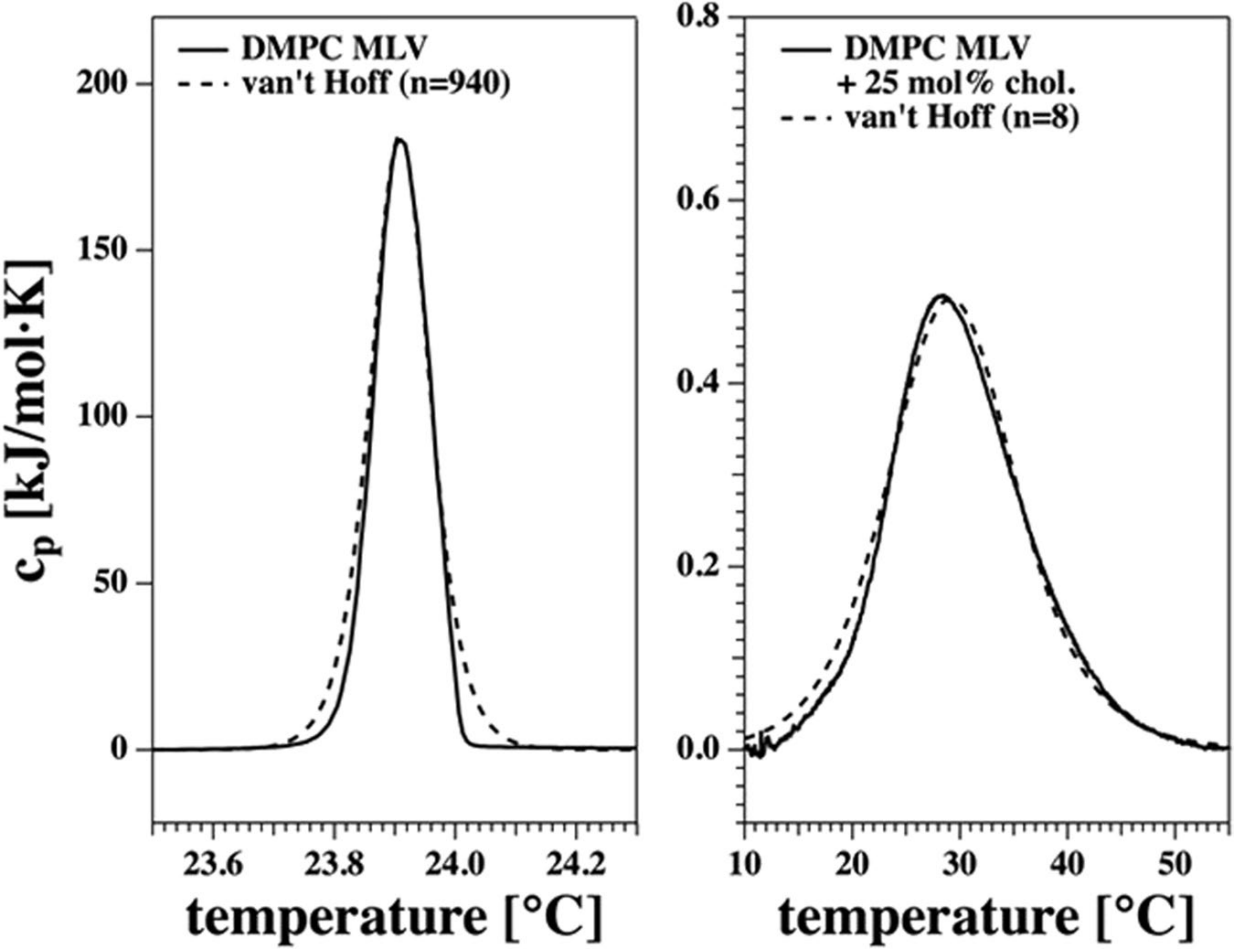
Heat capacity profiles from Fig. 1 for DMPC MLV (left) and DMPC + 25 mol% cholesterol (right). The dashed lines represent the calculated heat capacity profiles assuming a cooperative unit size of n = 940 for DMPC MLV and of n = 8 for the system in the presence of 25 mol% cholesterol.

To gain insight into the cooperative unit size of the lipids at the melting transitions, we went beyond such treatments and analysed the shape of the heat capacity profiles. Figure 6 shows the best description of the experimental profiles obtained from DSC of DMPC MLV and the DMPC + 25 mol% cholesterol mixture using eq. (13). The analysis yields cooperative unit sizes of n = 940 and n = 8, respectively. Assuming a lipid area of about 0.6 nm^2^ (DMPC in the fluid phase)^60^, we obtain a mean diameter of a cooperative unit of 27 nm at the c_p_-maximum of DMPC MLV, while it is about 2.5 nm in the mixture containing 25 mol% cholesterol. This gives an estimate for the characteristic length scale of the cooperative unit dominating the fluctuations to be probed and a clear hint that the correlation length is increasing in presence of a more pronounced phase transition.

The found value of 27 nm for DMPC MLVs coincides perfectly with similar values found in neutron diffraction experiments with ~24 nm^61^ probing the co-existence of gel and fluid lipid domains in this system. Toppozini *et al*.^62^ have reported a characteristic overall length scale of 10–20 nanometres for dynamic domains of cholesterol/DMPC patches, nearly an order of magnitude larger than the calculated cooperative unit of 2.5 nm in the mixture with 25 mol% cholesterol. On the other hand Armstrong *et al*.^63^ calculated a domain size of ~7 nm by neutron diffraction experiments of samples containing 32.5 mol%. This may indicate the presence of small fluctuating domains in such binary mixtures being potential nuclei of rafts in biological membranes as argued by Toppozini *et al*.^62^.

When one inserts now the heat capacities at maximum for the lipid preparations shown in Fig. 1, one obtains the relaxation times defined by eq. (15) and given in Table 4. They are about 22 seconds for pure DMPC MLV, and around 60 milliseconds in the presence of 25 mol% cholesterol. In a non-equilibrium thermodynamics treatment, relaxation timescales and fluctuation lifetimes are the same. Thus, PPC monitors fluctuation lifetimes on a timescale that is 10^7^–10^9^ times longer than the typical time-resolution of neutron scattering experiments. In contrary, neutron scattering monitors isomerizations within single lipids, which have a time scale in the range of nano-seconds^64^. Therefore, we confirm that fast timescales monitored by neutron scattering contain elements of the slow large-scale fluctuations, which would be impossible if the relaxation process was really single-exponential as stated in eq. (14). This fact is in line with the findings of Elamrani and Blume^65^, which have shown that the relaxation processes in lipid membranes close to transitions, as measured by light scattering after a pressure jump, are not well described by a single relaxation process and that fast processes exist, in agreement with the general statement that fluctuations exist at any scale close to a phase transition. Their data would be well described by a stretched exponential function, (~exp(−(t/τ)^α^), that extends down to very fast time scales. Similar effects were also found in proteins, were correlation of motions and possible linking of timescales from sub-ps to ns were experimentally established^47^. They are also in agreement with a recent publication by Hu *et al*.^66^, which used molecular dynamics simulations to show that motions in single proteins are self-similar and non-equilibrium. They established that the characteristic relaxation time for a distance fluctuation, such as inter-domain motion, is observation-time-dependent, increasing in a simple, power-law fashion. Comparison with single-molecule experiments suggests that such dynamical behaviour persists up to timescales approaching the *in vivo* lifespan of individual protein molecules. Following these arguments and combining the two methods presented here, we rationalise that the cooperative fluctuations of the membranes can be observed even at very short timescales as observed by incoherent neutron scattering that are faster than the relaxation processes of large domains. Therefore, our approach opens new possibilities to study membrane kinetics by the use of alternative techniques probing the extreme ends of the time scale, but which will finally give the same results.

**Table 4:** Experimental heat capacities at the maximum of the transition for DMPC MLV containing 0 mol%, 13 mol% and 25 mol% taken from Fig. 1.

	experimental c_p_ [kJ/mol·K]	calculated τ [s]
DMPC	184	22.1
DMPC + 13 mol% chol.	6.26	0.75
DMPC + 25 mol% chol	0.496	0.06

The relaxation times were calculated using eq. (15) assuming L = 7.35·10^8^ J·K/s·mol.

## Materials and Methods

### Differential Scanning Calorimetry (DSC) and pressure perturbation calorimetry (PPC)

DMPC and cholesterol were purchased from Avanti Polar Lipids (Alabaster, Alabama) and used without further purification (purity certified by the supplier >99%). DMPC/Cholesterol mixtures were prepared from chloroform stock solutions of the pure lipids. The chloroform solutions of the mixtures were dried under gentle flow of nitrogen, followed by a night in a vacuum desiccator to assure that no chloroform residuals remain in the samples. Finally, the dry lipid mixtures were hydrated with Millipore water above the melting temperature of the membranes while shaking the samples with a vortexer to obtain MLV.

A VP-DSC from MicroCal (Northampton, MA) equipped with a pressure perturbation unit was used. The DSC scan rate was 5 °C/hour. For pure DMPC we used samples containing 10 mM (=6.78 mg/ml) lipid. For the DMPC/cholesterol mixtures we used samples containing 30 mM (=20.34 mg/ml) DMPC. The pressure calorimeter, its design and the theory behind it was described in detail in ref.^7^. PPC relies on the above principles: At a given temperature, a small pressure perturbation is applied which leads to a change in enthalpy. The change is recorded and translated into an excess volume change. The experiment is then repeated at all temperatures. Both techniques probe the system as a whole.

The uncertainties in the determination of γ-values in Table 1 are originating from the subjectivity in scaling the heat capacity profiles Δc_p_ and the volume expansion coefficients α_V_ in Fig. 1 ^6^. The reproducibility of the heat capacity (Fig. 4) when iterating identical experiments is of the order of 1-2% and due to sample weighing and baseline subtraction (see Table 2 of ref.^6^). The c_p_ measurements and the volumetric experiments were done on the same samples.

### Elastic incoherent neutron scattering experiments

Samples were prepared as previously described^32^. Shortly, fully protonated DMPC powder was purchased from Avanti Polar Lipids (Alabaster, Alabama) and used as received (purity certified by the supplier > 99%). The cholesterol was purchased from Sigma-Aldrich® (St Louis, MO, USA). For the neutron experiments lipid powder was hydrated in a desiccator from a pure D_2_O atmosphere for at least two days at 40 °C^67^ in order to assure a fully hydrated sample. To permit a homogeneous pressure transmission, D_2_O was added after filling the pressure cell with the hydrated lipids. Before the neutron experiments, DSC of pure DMPC hydrated with H_2_O and D_2_O was measured. No differences in the transition temperature were detected (unpublished results).

The two DMPC/Cholesterol mixtures were prepared following the same protocol^31^ to obtain the two cholesterol concentrations of 25 mol% and 50 mol%. The lipids were mixed with the sterol in a chloroform solution and dried on the surface of an Erlenmeyer resulting in a thin layer of DMPC and cholesterol mixture. Adding heavy water to achieve the desired concentration and after 5 cycles of freezing-thawing-vortexing in a liquid nitrogen bath and in a water bath at 60 °C, MLV with a homogeneous concentration of DMPC and cholesterol were obtained. Both samples were at a final concentration of 100 mg/mL for a volume of about 1 mL.

EINS measurements as a function of temperature and pressure were performed on the thermal (λ = 2.23 Å) high-energy resolution backscattering spectrometer IN13^68^ (ILL, Grenoble, France), characterized by a very large momentum transfer range (0.2 < Q < 4.9 Å^−1^) with a nearly Q-independent energy resolution of 8 μeV full width half maximum. IN13, therefore, allows accessing the space and time windows of 1–30 Å and 0.1 ns, respectively. Fixed energy window (FEW) scans were recorded in a temperature range from 12 to 45 °C in 5 °C steps at 1, 300 and 600 bar. Each pressure was set with the help of the ILL high pressure equipment^69^ for neutron studies of biological samples in solution. The set-up consists of a cylindrical pressure cell made of a high-tensile aluminum alloy (7049-T6), a material which is mainly transparent for neutrons. The cell has an inner diameter of 6 mm and an outer diameter of 10 mm. Despite its thickness, the empty cell has a neutron transparency of about 92%. The wall thickness of 4 mm allows a pressure load up to 1.5 kbar. The sample volume was reduced to about 1 ml using a cylindrical aluminium insert of 4 mm diameter. The dimensions probed with this technique lie within the nanometre scale.

After 15 minutes of equilibration at the requested temperature set, typical counting times were between 3 and 7 h per temperature with an increasing counting time at higher temperature where the scattered intensity is reduced. For data correction also the empty high pressure cell, the cell filled with D_2_O at the same three pressure values as well as the cell filled with a vanadium sheet were measured. Data reduction was performed using the Large Array Manipulation Program (LAMP) available at the ILL^70^. Raw data were corrected by normalizing to the incident neutron flux, subtracting the background given by the spectrum of the D_2_O in the empty cell, and finally normalizing to a vanadium spectrum from which the empty cell was subtracted. The error bars were based on Poisson statistics applied to the raw data and the summed intensities. MSD were obtained according to eq. (17) (without any polydispersity and setting C_α_(τ) = 0). Then we fitted the MSD according to eq. (20) with a force constant defined by eq. (21) and the volume change as function of the temperature to get the thermal expansivity. In each case, the errors corresponded to fit errors including a weight for the experimental points.

### Theory

#### Calorimetry

The excess enthalpy ΔH of a melting transition is pressure dependent:

3 $${\rm{\Delta }}H(p)={\rm{\Delta }}E+p{\rm{\Delta }}V,$$

where ΔE is the excess internal energy difference between fluid and gel state, and ΔV is the excess volume change between the two states. Both ΔE and ΔV will be assumed to be independent of pressure. Therefore, the pressure dependence of the enthalpy can be determined if the excess volume ΔV is known.

The melting point T_m_ of a membrane is defined as the temperature where the free energy difference between fluid and gel phase is zero (i.e., equal amounts of gel and fluid lipids coexist):

4 $${\rm{\Delta }}G(p)={\rm{\Delta }}H(p)-{T}_{m}{\rm{\Delta }}S=0$$

where ΔS is the entropy difference between fluid and gel state. ΔS is assumed to be independent of pressure in first order approximation and called ΔS_0_ in the following, yielding

5 $${T}_{m}(p)=\frac{{\rm{\Delta }}H(p)}{{\rm{\Delta }}{S}_{0}}=\frac{{\rm{\Delta }}E}{{\rm{\Delta }}{S}_{0}}+p\frac{{\rm{\Delta }}V}{{\rm{\Delta }}{S}_{0}}.$$

Defining, $p\equiv {p}_{0}+{\rm{\Delta }}p$ with ${p}_{0}\equiv 1bar$, ${\rm{\Delta }}{H}_{0}\equiv {\rm{\Delta }}{H}_{0}({p}_{0})$ and ${T}_{m,0}={T}_{m}({p}_{0})={\rm{\Delta }}{H}_{0}/{\rm{\Delta }}{S}_{0}$ we can rewrite eq. (5) as

6 $${T}_{m}(p)={T}_{m,0}+{\rm{\Delta }}p\frac{{\rm{\Delta }}V}{{\rm{\Delta }}{S}_{0}}={T}_{m,0}(1+\frac{{\rm{\Delta }}p{\rm{\Delta }}V}{{\rm{\Delta }}{H}_{0}})$$

or

7 $${T}_{m}(p)={T}_{m,0}\,(1+\gamma {\rm{\Delta }}p),$$

where γ = ΔV/ΔH_0_ is a pressure‐independent parameter. When γ is known, the pressure dependence of the transition temperature can be determined.

ΔH_0_, ΔS_0_ and ΔV are all temperature dependent functions. ΔH_0_ can easily be determined by determining the excess heat capacity in calorimetric experiments through

8 $${\rm{\Delta }}{c}_{p}(T)=\frac{d{\rm{\Delta }}{H}_{0}(T)}{dT};\,{\rm{\Delta }}{H}_{0}(T)={\int }_{{T}_{o}}^{T}{\rm{\Delta }}c{}_{p}dT.$$

The excess volume change ΔV can be measured in PPC through the volume expansion coefficient, α(T), defined as

9 $$\alpha (T)={(\frac{d{\rm{\Delta }}V(T)}{dT})}_{p};\,{\rm{\Delta }}V(T)={\int }_{{T}_{o}}^{T}\alpha dT,$$

which can be compared with the excess heat capacity. The specific volume expansion coefficient is the normalized quantity

10 $${\alpha }_{V}(T)=-{(\frac{{\rm{\Delta }}{\rm{\Delta }}V(T)}{\langle V\rangle {\rm{\Delta }}T})}_{p}.$$

It can be determined in PPC using the relation

11 $${\alpha }_{V}(T)=-\frac{{\rm{\Delta }}Q}{T\langle V\rangle {\rm{\Delta }}p},$$

where ΔQ is the absorption of heat by a sample exposed to a pressure change Δp. This equation is related to the Clausius‐Clapeyron relation.

Lipids melting transitions are cooperative events. This means that many lipids change their state at the same time in a collective manner. In a simplifying way one may assume that *n* lipids undergo a transition at the same time. Assuming further a two-state transition between solid and liquid membrane, one can describe the melting by the

12 $$gel\,\mathop{\longleftrightarrow }\limits^{{\rm{\Delta }}{H}_{vH},{\rm{\Delta }}{S}_{vH}}\,{\rm{fluid}}$$

equilibrium, where the van’t Hoff enthalpy is ∆H_vH_ = *n*·∆H and the associated entropy change is ∆S_vH_ = *n*·∆S. *n* is usually called cooperative unit size, ∆H is the excess enthalpy per mole of lipid, and ∆S is the excess entropy per mole of lipid. The heat capacity profiles assume the form^28^

13 $${c}_{p}=(\frac{K}{{(1+K)}^{2}})\frac{n\,{\rm{\Delta }}H}{R{T}^{2}},\,{\rm{where}}\,K=\exp (-\frac{n({\rm{\Delta }}H-T{\rm{\Delta }}S)}{RT}),$$

R is the gas constant and K the equilibrium constant for n lipids. Lipids’ pre-transitions are linked to the formation of periodic membrane ripples and are much less cooperative than the main transition^71^, therefore they are not considered here.

Time scales can be measured in perturbation experiments or by analysing the time scale of fluctuations. In pressure calorimetry of lipid membranes, heat is released after a pressure perturbation of the membrane. Most of that heat change can be described by a single exponential function^72,73^

14 $${\rm{\Delta }}H(t)={\rm{\Delta }}{H}_{0}\,{\exp }\,(-\frac{t}{\tau })$$

where the relaxation time τ is given by

15 $$\tau =-\frac{{T}^{2}}{L}{c}_{p}.$$

L is a phenomenological coefficient, which is similar for many different lipid systems^72,73^. Its value is 7.35·10^8^ J·K/s·mol for DMPC MLV.

### Elastic Incoherent Neutron Scattering

EINS is largely dominated by the signal from hydrogen atoms, because their incoherent scattering cross section is much higher than that for any other type of atom present in biological samples^74^. The averaged atomic motion of hydrogen atoms, which are mostly homogeneously distributed in biological macromolecules, informs thus over local atomic vibrations and translations of static positions.

Based on van Hove’s formalism^75^ calculated for the pair correlation function and taking into account the time resolution τ of the instrument, it can be shown that EINS data is related to the normalized elastic intensity *I* through^38^

16 $${S}_{inc}^{el}(Q)\approx I(Q,\tau )=\sum _{\alpha =1}^{N}{x}_{\alpha }\exp \,\{-\frac{1}{3}\,{Q}^{2} < {{r}_{\alpha }}^{2} > [1-{C}_{\alpha }(\tau )]\},$$

where x_α_ (with $\sum _{\alpha =1}^{N}{x}_{\alpha }=1$) is the fraction of particles experiencing the same dynamics in the potential V_α_(**r**), $< {{r}_{\alpha }}^{2} >$ the equilibrium mean square displacement and C_α_(τ) accounts for the stationary position relaxation function (for more details see ref.^38^). The MSD <R^2^ (T)>, which take into account fluctuations of all particles in the system within τ, are given by

17 $$ < {R}^{2}(T) > \,=\,{-3\frac{d\mathrm{ln}{S}_{inc}^{el}(Q)}{d{Q}^{2}}|}_{Q=0}=\sum _{\alpha =1}^{N}{x}_{\alpha }\, < {{r}_{\alpha }}^{2} > [1-{C}_{\alpha }(\tau )].$$

It signifies that C_α_(τ) is a constant for a given instrument and rescales the observed MSD, so that one can assume without loss of generality that C_α_(τ) = 0. Within the Gaussian approximation^32,33^, which supposes a Gaussian distribution of the particles’ motions around their rest position, and neglecting quantum effects due to zero-point fluctuations, this expression can be transformed into^38^

18 $$ < {R}^{2}(T) > =\sum _{\alpha =1}^{N}{x}_{\alpha }\{{\varphi }_{\alpha }(T)\frac{{k}_{B}T}{{k}_{1\alpha }}+[1-{\varphi }_{\alpha }(T)]\frac{{k}_{B}T}{{k}_{2\alpha }}\},$$

where *k*_*B*_ is the Boltzmann constant. Such a model assumes that the atomic motions in a biomolecule, e.g. a protein or a lipidic system, are essentially dominated by two states characterized by separate movements - fluctuations around the equilibrium positions and larger agitations of the particle within a restricted volume (cage) formed by neighbouring molecules. k_1__α_ and k_2__α_ are force constants according to^76^. ϕ_α_ is defined by:

19 $${\varphi }_{\alpha }=\frac{1}{1+{e}^{-\beta {\rm{\Delta }}G}}\,{\rm{with}}\,{\rm{\Delta }}{G}_{\alpha }={\rm{\Delta }}{H}_{\alpha }-T{\rm{\Delta }}{S}_{\alpha }.$$

β^−1^ = k_B_T is the thermal energy, and ΔG_α_, ΔH_α_ and ΔS_α_ are the differences between the corresponding thermodynamic quantities for the large and small cages. As stated in eq. (3), ΔH_α_ is pressure dependent, i.e. *ΔH*_α_ = *ΔE*_*α*_ + *p ΔV*_*α*_.

**Figure 7:**
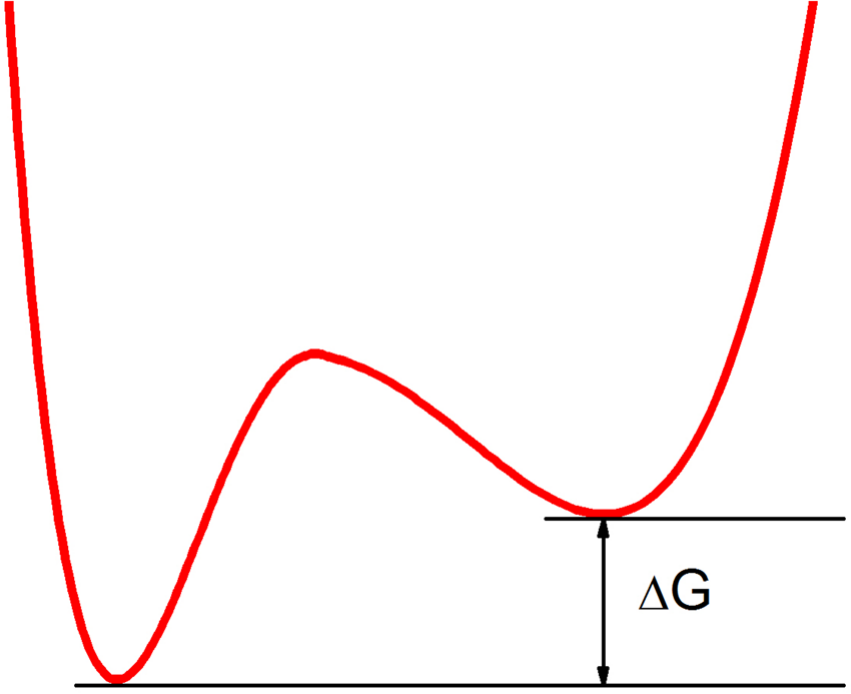
Sketch of the two potentials corresponding to the smaller and larger fluctuations. The width of each well is a qualitative indication of the root-mean-square fluctuation, $\sqrt{\langle {u}^{2}\rangle }$, and of the entropy of the corresponding state.

ϕ_α_ represents the probability to find a particle in a given conformational cage at lower temperature corresponding to the smaller kind of movement, and (1 − ϕ_α_) the complementary probability to find it in the higher entropy state corresponding to the larger fluctuation type of motion as presented in the sketch (see Fig. 7). Such view is similar to the double-well potential model introduced by Doster *et al*.^53^ to account for non-Gaussian motions in myoglobin at temperatures above 200 K, but it holds for any transition assuming only two possible states.

If we assume that all atoms undergo the transition in a cooperative manner, it would be equivalent to a van’t Hoff analysis considering only two states. ΔG represents thus the mean free energy difference ${\rm{\Delta }}G={\sum }_{\alpha =1}^{N}{x}_{\alpha }{\rm{\Delta }}{G}_{\alpha }$, where ΔG_α_ is the molar enthalpy. It is equivalent to a mean field approach, where the two potentials represented in Fig. 7 are generated by the average interactions of all neighbouring particles and separated by the energy quantity given by the mean free energy difference. Similar to eqs (4) and (5) the melting temperature T_m_ is defined where both fluctuations are equivalent and therefore ΔG (T_m_) = 0.

To extract the energy states from the neutron data, a simplified empiric, though realistic two-state model was used by Bicout and Zaccai^38^ to study its applicability

20 $$\langle {u}^{2}(T)\rangle =\varphi (T,p)\,\frac{{k}_{B}T}{{k}_{1}}+(1-\varphi (T,p))\,\frac{{k}_{B}T}{{k}_{2}},$$

where $\varphi ={\sum }_{\alpha =1}^{N}{x}_{\alpha }{\varphi }_{\alpha }$ and k_1_ and k_2_ are effective force constants which are functions of x_α_, ϕ_α_, k_1α_ and k_2α_ and thus functions of temperature and pressure, and the more general model eq. (18) with two dynamically non-equivalent fractions of particles to analyse MSD data from the purple membrane of *Halobacterium salinarium*. Their fits resulted in one population with same parameters as when used only one fraction, larger population and another fraction, smaller population with different parameters, comparable to the first two contributions from a Taylor series development. Therefore, it seems reasonable to fit the data in a first approximation with k_1_ and k_2_ as independent of temperature and pressure. The temperature and pressure dependences are then only contained in the fraction ϕ_α_ defined by eq. (19).

According to the theoretical introduction above, it is therefore a valid approach to extract thermodynamic parameters from neutron data. Using for instance the double-well potential proposed by Doster *et al*.^53^ and a van’t Hoff plot of the logarithm of the ratio of the two probabilities of occupancy of well 1 and 2 against 1/T, the intercept and slope of the resulting curves permit to extract the excess quantities of ΔG, ΔH and ΔS with fair accuracy (see for example^77^). In the present study we have demonstrated that the thermodynamic quantities obtained from neutron data are in agreement with results from DSC and PPC, although the underlying time scales are completely different. This fact is theoretically ensured by the presence of statistical fluctuations at any scale due to the phase transition, but the mean field approach has to be validated. In addition, pressure has been taken into account for the treatment, what was generally not done so far in neutron experiments performed.

It may look challenging to compare thermodynamic quantities (energies) extracted from DSC and from EINS, but they turn out to be identical as both methods investigate the same system changes and transition using different probe: heat for the DSC and the thermal fluctuations of motion (as a manifestation of the heat) for EINS. In both cases the changes correspond to thermodynamic differences between the gel and fluid state of the lipids, which can be associated for EINS to the small and large cages limiting molecules’ motions, therefore to excess quantities. The main difference consists in the time scales: DSC measures a heat flow over seconds to minutes, whereas the neutron scattering technique gives access to a time window of about 100 ps while the sample is heated up continuously. Even if the relative low neutron flux does not allow an as fine temperature resolution as DSC, the measurement time, the larger sample volume and the temperature ramp during the EINS permit a direct correlation between neutron and calorimetry data.

As HHP experiments can only be done in solution to ensure a homogeneous transmission of the hydrostatic pressure and at temperatures larger than 4 °C in order to avoid ice formation, only the temperature range above this temperature has been used in the fitting. We used a single effective force constant defined as

21 $$\frac{1}{\langle {k}_{eff}(T,p)\rangle }=\frac{\varphi (T,p)}{{k}_{1}}+\frac{(1-\varphi (T,p))}{{k}_{2}},$$

where the part corresponding to lower temperature, ϕ(T, p)/k_1_, which is varying only very slowly, was approximated by a constant value. We introduce it as an effective force constant, following Zaccai’s suggestion^76^ with respect to the temperature dependence, as it is valid and constant for a certain scale or range of a parameter, only. On larger scales, it depends explicitly on temperature and pressure and also on experimental conditions like hydration or pH value or even the instrumental resolution giving access to more or less motions within the corresponding time window (see for instance the different slopes of the MSD of heparan sulfate when measured on three different spectrometers described in ref.^78^).

As fluctuations in temperature and volume are statistically independent^79^, volume changes −ΔV induced by HHP application can be obtained through

22 $${-{\rm{\Delta }}V|}_{T}=RT{\frac{\partial \mathrm{ln}(K)}{\partial P}|}_{T},$$

where K corresponds to the equilibrium constant of a two-state system (ambient pressure and HHP) defined as $K={e}^{-({\rm{\Delta }}E+p{\rm{\Delta }}V-T{\rm{\Delta }}S)/{k}_{B}T}$. The pressure dependence of the thermal expansivity Δα = ∂ΔV/∂T)|_P_ can thus be obtained by fitting ΔV against temperature.
